# ADDRESSING DISPARITIES IN ADRD PREVALENCE AND MORTALITY RATES BETWEEN RURAL AND URBAN COMMUNITIES

**DOI:** 10.1093/geroni/igae098.1822

**Published:** 2024-12-31

**Authors:** Nasim Ferdows

**Affiliations:** Northeastern University, Boston, Massachusetts, United States

## Abstract

Rising life expectancy has led to an increase in age-related diseases, such as Alzheimer’s disease and related dementia (ADRD). The rapid growth of the rural older population may result in higher rates of dementia in these communities. However, rural residents face disparities in healthcare access, potentially leading to an underestimation of ADRD prevalence in claims-based studies requiring medical assessment. This study employs two approaches to examine rural and urban differences in ADRD prevalence and mortality rates. First, analyzing national county-level data from the CDC Wonder database from 2010 to 2021, we found higher ADRD mortality rates in rural areas, though not statistically significant. Second, using Health and Retirement Study data, a nationally representative sample of US older adults, and a 20% sample of Medicare claims, we observed higher dementia prevalence in rural areas based on cognitive tests in the HRS, which are free from clinical assessment. This contrasts with lower prevalence rates based on diagnosis codes in claims data for rural areas. The decline in prevalence rates based on diagnosis codes was also more pronounced in urban areas. The decline in prevalence rates based on diagnosis codes was also sharper in urban areas. These findings suggest that claims-based studies may underestimate dementia rates in rural areas due to limited healthcare access. Addressing these disparities is critical for providing adequate care and support to individuals with ADRD in rural communities.

